# Neuroprotective Effects of Tetrahydrocurcumin against Glutamate-Induced Oxidative Stress in Hippocampal HT22 Cells

**DOI:** 10.3390/molecules25010144

**Published:** 2019-12-30

**Authors:** Chang-Hyun Park, Ji Hoon Song, Su-Nam Kim, Ji Hwan Lee, Hae-Jeung Lee, Ki Sung Kang, Hyung-Ho Lim

**Affiliations:** 1 College of Korean Medicine, Gachon University, Seongnam 13120, Korea; chpark104@naver.com (C.-H.P.); jhsong.john@gmail.com (J.H.S.); kleert26@gmail.com (J.H.L.); 2 Natural Products Research Institute, Korea Institute of Science and Technology Gangneung 25451, Korea; snkim@kist.re.kr; 3 Department of Food and Nutrition, Gachon University, Seongnam 13120, Korea; skysea@gachon.ac.kr or

**Keywords:** glutamate, oxidative stress, mitogen-activated protein kinase, Ca^2+^, tetrahydrocurcumin, HT22 cells

## Abstract

In the central nervous system, glutamate is a major excitable neurotransmitter responsible for many cellular functions. However, excessive levels of glutamate induce neuronal cell death via oxidative stress during acute brain injuries as well as chronic neurodegenerative diseases. The present study was conducted to examine the effect of tetrahydrocurcumin (THC), a major secondary metabolite of curcumin, and its possible mechanism against glutamate-induced cell death. We prepared THC using curcumin isolated from *Curcuma longa* (turmeric) and demonstrated the protective effect of THC against glutamate-induced oxidative stress in HT22 cells. THC abrogated glutamate-induced HT22 cell death and showed a strong antioxidant effect. THC also significantly reduced intracellular calcium ion increased by glutamate. Additionally, THC significantly reduced the accumulation of intracellular oxidative stress induced by glutamate. Furthermore, THC significantly diminished apoptotic cell death indicated by annexin V-positive in HT22 cells. Western blot analysis indicated that the phosphorylation of mitogen-activated protein kinases including c-Jun N-terminal kinase, extracellular signal-related kinases 1/2, and p38 by glutamate was significantly diminished by treatment with THC. In conclusion, THC is a potent neuroprotectant against glutamate-induced neuronal cell death by inhibiting the accumulation of oxidative stress and phosphorylation of mitogen-activated protein kinases.

## 1. Introduction

Neurodegenerative diseases have no treatment currently available, and the principal goals for neurodegeneration care are early diagnosis, physical health, optimizing, identifying and treating abnormal behavioral and psychological symptoms. Glutamate is a neurotransmitter that plays critical roles in various physiological as well as pathological brain functions [1]. Under normal conditions, glutamate is responsible for cell survival, migration, and differentiation during brain development, while an excessive amount of glutamate leads to neuronal cell death through oxidative stress or excitotoxicity [1,2]. Reactive oxygen species (ROS) is one major cause of neuronal cell death in chronic neurodegenerative diseases [3,4]. Earlier studies showed that an excessive release of glutamate in the extracellular region provoked the accumulation of intracellular ROS by depletion of intracellular glutathione levels through blocking cysteine uptake [5]. The presence of an antioxidant, such as flavonoids or *N*-acetylcysteine, strongly prevented ROS-induced neuronal cell death [6,7].

The use of natural antioxidants for scavenging free radicals and maintaining homeostasis contributes to alleviating neuronal dysfunction [8]. Typically, natural compounds are multiple-target molecules found mainly in microorganisms and plants and exhibit strong antioxidant activity [8,9]. Phenolic compounds from natural sources also exhibit various beneficial effects in cancer, inflammation, and neurodegenerative disorders [9]. This broad spectrum of biological or pharmacological activities has made phytochemicals suitable candidates for treating multifactorial diseases, such as neurodegenerative diseases [10,11]. However, there are also several concerns regarding their poor bioavailability, stability and safety, which must be resolved before effective therapeutics can be developed. 

Mitogen-activated protein kinases (MAPKs), known as a family of serine/threonine protein kinases, are well-identified biological molecules involved in glutamate-induced oxidative neuronal cell death. MAPKs are related to multiple cellular functions including differentiation, proliferation, cell survival, inflammation, and cell death [12]. The presence of excessive glutamate triggers ROS, an increase in intracellular calcium ion concentration, and the activation of MAPKs, including phosphorylation of c-Jun N-terminal kinase (JNK), extracellular signal-regulated kinase (ERK), and p38, which are responsible for neuronal cell death in both primary neuronal cultures and neuronal cell lines [13]. Recently, we found that phytochemicals, such as chebulinic acid and casuarinin, markedly prevented ROS, elevation of intracellular calcium level, and phosphorylation of MAPKs by excessive glutamate-induced HT22 cell death [14,15]. Therefore, the inhibition of MAPK phosphorylation is a key mechanism of neuroprotective compounds among the biological molecules involved in glutamate-induced oxidative stress. 

Tetrahydrocurcumin (THC) is a major secondary metabolite of curcumin known as a major bioactive compound of *Curcuma longa* (turmeric) [16]. Curcumin is typically metabolized in the intestine to THC which has strong antioxidant activity [17]. THC is stable at a wide range of pH, and can be easily absorbed through the gastrointestinal tract. Furthermore, THC also plays a critical role in biological effects of curcumin [17]. It has been reported that THC shows anti-inflammatory, anticarcinogenic activities, and neuroprotective effects [18,19]. However, the effect of THC needs to be clarified on glutamate-related neuronal cell death. Therefore, the present study was carried out to demonstrate the possible effect and the protective mechanism of THC on glutamate-mediated neuronal cell death.

## 2. Results and Discussion

**Figure 1:**
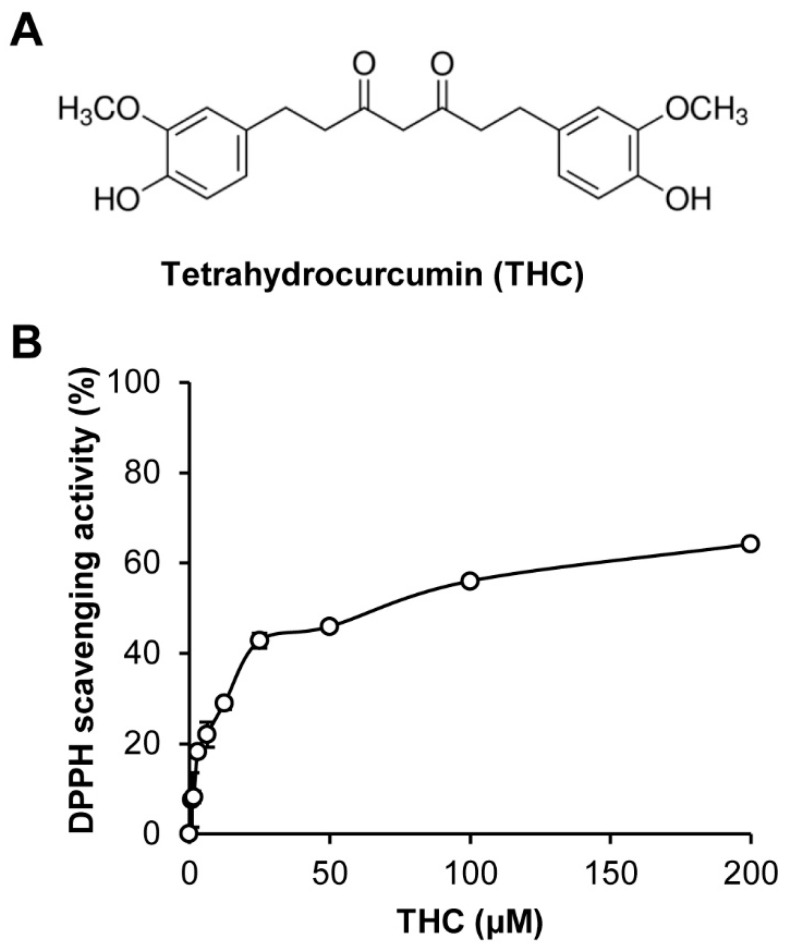
Tetrahydrocurcumin (THC) possessed antioxidative activity. (**A**) Chemical structure of THC prepared from curcumin isolated from *Curcuma longa* (Turmeric). (**B**) The bar graph represents DPPH scavenging activity of THC.

It is well known that natural products including plant materials contain several antioxidative compounds. Curcumin, a major bioactive compound of *Curcuma longa* (turmeric), is typically metabolized in the intestine to THC as a major secondary metabolite and has strong antioxidant activity (Figure 1A). We also confirmed the antioxidant activity of THC through an in vitro 1,1-diphenyl-picryl hydrazyl (DPPH) radical scavenging assay, which is used to evaluate the antioxidant effects. Consistently, our results showed that THC had strong DPPH scavenging activity (Figure 1B). 

**Figure 2:**
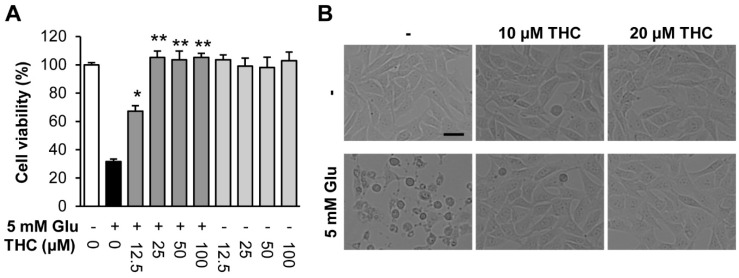
Tetrahydrocurcumin (THC) prevented glutamate-induced HT22 cell death. (**A**) Cell viability was measured using a CyTox assay kit 24 h after treatment with 5 mM glutamate with or without THC. Bars denote the percentage of cell viability (mean ± S.E.M., * *p* < 0.05 and ** *p* < 0.001 compared to glutamate-treated cells). (**B**) Microscopic images were obtained after exposure of HT22 cells to glutamate for 24 h (scale bar, 50 μm).

Several studies have demonstrated that curcumin and THC have a strong antioxidant effect and prevent neuronal cell death in traumatic brain injuries [20,21]. Thus, THC may attenuate neuronal cell death induced by glutamate in HT22 cells. To assess the neuroprotective effects of THC on glutamate-induced oxidative stress, we incubated HT22 cells with 5 mM glutamate in the absence or presence of THC for 24 h. We found that glutamate decreased cell viability, while THC increased cell viability significantly at concentrations of 10 and 20 µM compared to that in glutamate-treated cells (Figure 2A). Morphologically, THC almost completely inhibited HT22 cell death induced by glutamate (Figure 2B). Our data suggest that THC is a potent neuroprotectant against glutamate-induced HT22 cell death in neurodegenerative diseases.

**Figure 3:**
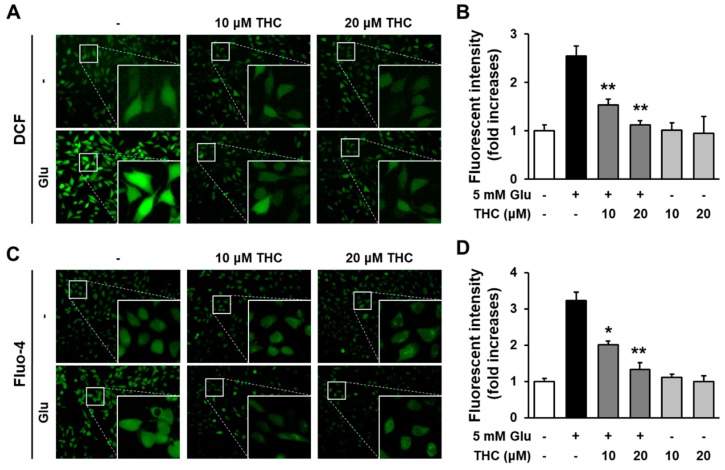
THC diminished the increase in intracellular ROS and Ca^2+^ via its antioxidant activity. (**A**) Cells were treated with 5 mM glutamate with or without 10 or 20 μM THC for 8 h and stained with H2DCF-DA. Green indicates DCF fluorescence (20×). (**B**) Bars denote the ROS levels measured by fold-increases in the fluorescent intensity of DCF (** *p* < 0.001 compared to glutamate-treated cells). (**C**) Cells were treated with 5 mM glutamate with or without 10 or 20 μM THC for 8 h and stained with Fluo-4 AM for an additional 30 min. Green fluorescence indicates DCF active cells (20×). (**D**) Bars denote the levels of [Ca^2+^]_i_ measured by fold-increases in the fluorescent intensity of Fluo-4 (* *p* < 0.05 and ** *p* < 0.001 compared to glutamate-treated cells).

Glutamate induces oxidative stress-mediated neuronal cell death in both acute brain injuries as well as neurodegenerative disease [5]. Because oxidative stress is a major event during neuronal cell death, preventing ROS is a possible strategy for attenuating neuronal cell death. Thus, we investigated whether THC could reduce glutamate-induced accumulation of intracellular ROS in HT22 cells. The cells were exposed to 5 mM glutamate with 10 or 20 µM THC for 8 h and then stained with H2DCF-DA to evaluate intracellular ROS levels. Our results showed that THC markedly prevented the accumulation of intracellular ROS increased by glutamate treatment, and quantitative analysis showed that treatment with glutamate in HT22 cells increased the ROS production measured by fluorescent intensity of DCF to 2.54-fold, but the fluorescent intensity significantly reduced by THC (Figure 3A,B). Previously, it has been suggested that intracellular Ca^2+^ ([Ca^2+^]_i_) is a characteristic of neuronal cell death by glutamate-induced oxidative stress [22,23]. Therefore, we also assessed the levels of [Ca^2+^]_i_ using Fluo-4 AM, a membrane-permeable fluorescent indicator for Ca^2+^. Our results showed that THC also prevented the glutamate-triggered elevation of [Ca^2+^]_i_ from 3.22-fold increases in glutamate-treated cells to 2.0- or 1.33-fold in 10 or 20 μM THC-treated cells, respectively (Figure 3C,D). Taken together, these results suggest that THC can protect HT22 cell from glutamate toxicity through the inhibition of oxidative stress and the increase in [Ca^2+^]_i_.

**Figure 4:**
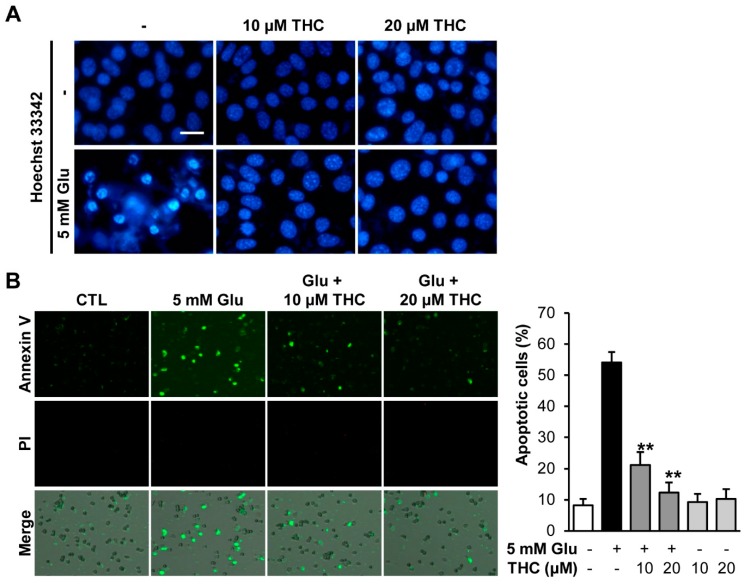
THC blocked glutamate-induced apoptotic cell death in HT22 cells. (**A**) Cells were treated with 5 mM glutamate with or without 10 or 20 μM THC for 12 h and stained with Hoechst 33342. (**B**) We also performed Tali-image based analysis to identify the percentage of apoptotic cells. Merged images (bottom) were combined with bright field (gray; not shown), annexin V (green; top), propidium iodide (red; middle) images. The bars denote the percentages apoptotic cells determined by annexin V-positive cells (** *p* < 0.001 compared to glutamate-treated cells).

Glutamate is known to induce both necrotic and apoptotic cell death. Previously, glutamate was shown to induce apoptotic cell death during early stages and necrotic cell death at a later time [24,25]. Therefore, we mainly focused on early apoptotic cell death induced by glutamate. To investigate the effect of THC on glutamate-induced apoptosis in HT22 cells, cells were treated with 5 mM glutamate in the presence and absence of 10 or 20 μM THC for 12 h. We first stained HT22 cells with Hoechst 33342 to see a chromatin condensation, which is a morphological feature of apoptotic cell death [26]. The result showed that the chromatin condensation was observed in glutamate-treated HT22 cells, while THC completely prevented those effects of glutamate (Figure 4A). In addition, we quantitatively analyzed the proportion of apoptotic cell population. HT22 cells were stained with propidium iodide (PI) and annexin V and quantitatively analyzed using image-based cytometric analysis. Our results showed that glutamate significantly increased the percentage of annexin V-positive cells, indicating apoptotic cells, whereas PI-positive cells were not detected (Figure 3A). This suggested that most HT22 cell death caused by glutamate occurred through the apoptotic pathway. However, THC markedly reduced the percentage of annexin V-stained cells (Figure 4B). Quantitative analysis results showed that 54.12% of glutamate-treated cells were annexin V-positive, while only 21.22% and 12.35% annexin V-positive cells were observed among glutamate-treated cells treated with 10 and 20 μM THC, respectively (Figure 3B). This shows that THC significantly reduced the number of apoptotic cell deaths after treatment with glutamate in HT22 cells. 

**Figure 5:**
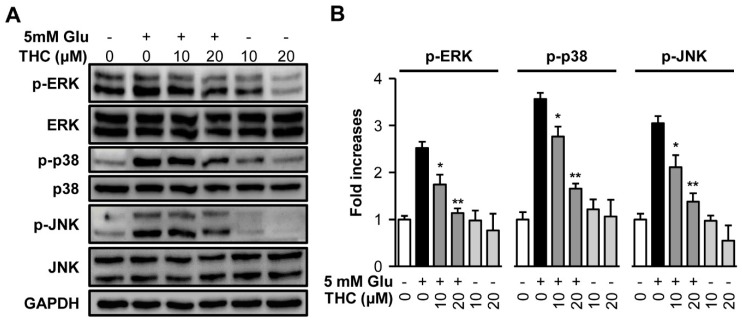
THC blocked phosphorylation of mitogen-activated protein kinases (MAPKs) induced by glutamate in HT22 cells. (**A**) HT22 cells were treated with 5 mM glutamate with or without 10 or 20 µM THC for 8 h. Western blot analysis were performed with specific antibodies for p38, p-p38, ERK, p-ERK, JNK, p-JNK, and GAPDH. (**B**) Immunoreactive bands were quantitatively determined using ImageJ software. The bars denote the fold increases compared with control (mean ± S.E.M, * *p* < 0.05 and ** *p* < 0.001 compared to glutamate-treated cells).

Inhibition of MAPK phosphorylation is a protective mechanism against glutamate-induced neuronal cell death [27]. MAPK activation is responsible for cell survival as well as death. It has been also reported that preventing the intracellular ROS blocked the phosphorylation of MAPKs and cell death induced by hydrogen peroxide activation, indicating the involvement of ROS-mediated phosphorylation of MAPKs in neuronal cell death [12,27,28]. In addition, treatment with a specific inhibitor of ERK, U0126, prevented glutamate-induced phosphorylation of ERK in primary cortical neurons and HT22 cells [12]. Therefore, we examined whether THC blocked MAPK activation. We treated HT22 cells with 5 mM glutamate and 10 or 20 μM THC for 8 h and performed Western blot analysis to detect MAPKs phosphorylation. The result showed that glutamate increased the activation of ERK, JNK, and p-38 by 2.52-, 3.57-, and 3.04-fold, respectively. In contrast, THC significantly diminished the phosphorylation of MAPKs induced by glutamate to control levels (Figure 5A,B). These results suggest that the inhibition of phosphorylation of MAPKs is a molecular mechanism of the THC-mediated neuroprotective effect against HT22 cell death induced by glutamate. 

The present study demonstrated that THC strongly prevented HT22 cell death induced by glutamate. Its protective mechanisms occur through inhibition of accumulation of intracellular ROS as an antioxidant property, blockage of MAPK activation, and prevention of apoptotic cell death of HT22 cells by glutamate. Therefore, THC may be useful as a potent neuroprotective metabolite of curcumin against glutamate-mediated neuronal death. However, as is the case with most natural products, poor oral bioavailability limits the therapeutic effects of phytochemicals in vivo. In addition to the problems regarding bioavailability, the results of the cell experiment may not be reflected at the organism level. An important direction for future investigation is the further optimization of the bioavailability of natural products in vivo. However, natural products are relatively safe and exhibit various beneficial effects. Therefore, combinatorial strategies which target multiple mechanisms, such as reducing oxidative stress and increasing anti-inflammation effects, may offer a better chance for clinically meaningful prevention. In addition, extensive efforts should be simultaneously made to solve challenges such as bioavailability, optimization of the lead material, and securing efficacy in clinical trials.

## 3. Materials and Methods

### 3.1. Preparation of Tetrahydrocurcumin

Tetrahydrocurcumin (THC) was prepared from curcumin as reported previously [17,18]. In brief, curcumin was isolated from the powder of turmeric, *Curcuma longa*. The isolated curcumin was then converted to tetrahydrocurcumin (THC) by hydrogenation reaction with 10% PtO_2_ as the catalyst. After hydrogenation, THC was purified by preparative HPLC, and identified by using NMR and MS spectra.

### 3.2. 1,1-Diphenyl-2-Picrylhydrazyl (DPPH) Radical Scavenging Activity

Each of the concentrations of THC were mixed with an equal volume of DPPH solution (Sigma, St. Louis, MO, USA). After incubation for 30 min at 25 °C, the absorbance value at 540 nm was measured using an E-Max microplate reader.

### 3.3. Cell Culture and Treatment

A murine hippocampal cell line, HT22 cells, were grown in Dulbecco’s modified Eagle’s medium (DMEM; Corning, Manassas, VA, USA) supplemented with 10% fetal bovine serum (Atlas, Fort Collins, CO, USA) and antibiotics (streptomycin/penicillin; Gibco, Grand Island, NY, USA). HT22 cells were maintained at 37 °C in a humidified incubator supplied with 5% CO_2_ [29]. The experimental conditions containing incubation times were determined according to the results of measuring each marker at various time points [15].

### 3.4. Measurement of Cell Viability

To assess the cell viability, we used MTT assay kit (EZ-CyTox; Daeil Lab Service, Seoul, Korea). HT22 cells were plated on 96-well plates at a density of 1 × 10^4^ per well and incubated for 24 h to adhere. The cells were then exposed to 5 mM glutamate for 24 h with the indicated concentrations of THC. Cells were then added with 10 µL of Ez-CyTox reagent followed by incubating the cells for 30 min. Absorbance values at 450 nm were obtained using an E-Max microplate reader (Molecular Devices, Sunnyvale, CA, USA). The viability of cells was represented by comparing the percentage of viability of the control group [30].

### 3.5. Nuclear Staining

HT22 cells were plated to 6-well plates and stained with Hoechst 33342 (Sigma) as reported previously [31]. After treatment with glutamate for 12 h, cells were incubated with 10 μM of Hoeschst 33342 for 10 min to verify the chromatin condensation. Fluorescent images with nuclear condensation were obtained using a fluorescent microscope (IX50).

### 3.6. Measurement of Intracellular ROS

Intracellular ROS levels were measured using 2′,7′-dichlorofluorescin diacetate (H2DCFDA; Sigma) [32]. Cells were exposed to 5 mM glutamate for 8 h and then incubated with 10 µM H2DCFDA for 30 min. A fluorescent intensity of DCF as an intracellular ROS level were measured using a fluorescent microplate reader (SPARK 10M; Tecan, Männedorf, Switzerland) at 495/517 nm (ex/em). Fluorescent images were then prepared using a fluorescent microscope (IX50; Olympus, Tokyo, Japan).

### 3.7. Fluo-4 Staining

To evaluate the levels of intracellular Ca^2+^, cells were treated with 5 mM glutamate for 8 h with or without THC. The cells were treated with 2 µM Fluo-4 AM (Invitrogen, Eugene, OR, USA) 30 min before imaging. Fluorescent images were acquired using a fluorescent microscope equipped (IX50; Olympus) and analyzed quantitatively using ImageJ software (National Institute of Health). 

### 3.8. Western Blot Analysis

After the exposure of HT22 cells to 5 mM glutamate for 8 h, cells were harvested and lysed with RIPA buffer (Cell Signaling, Danvers, MA, USA). The same amounts of proteins were electrophoresed using SDS-polyacrylamide gel and then transferred to a polyvinylidene difluoride membrane (Merck Millipore, Darmstadt, Germany) [33]. The membranes were incubated with 5% skim milk in tris-buffered saline containing 0.1% tween-20 (TBST) to block the nonspecific bindings. The membranes were then incubated with primary antibodies for c-Jun N-terminal kinase (JNK), phospho-JNK, extracellular signal-regulated kinase (ERK), phospho-ERK, p38, phospho-p38, and glyceraldehyde 3-phosphate dehydrogenase (GAPDH) (Cell Signaling) for 1 h at 25 °C. The membranes were then incubated with secondary antibodies (Cell Signaling). To visualize the immunoreactive bands, the membranes were reacted with SuperSignal West Femto Maximum Sensitivity Substrate (Thermo Scientific, Rockford, IL, USA).

### 3.9. Quantification of Apoptotic Cells

The apoptotic cells were measured using a Tali Image-Based Cytometer (Invitrogen) [34]. In brief, cells were harvested after the treatment of 5 mM glutamate for 12 h. The cells were treated with annexin-binding buffer and labeled with AlexaFluor 488-conjugated annexin V for 20 min. The cells were then stained further with propidium iodide. The stained images were obtained and analyzed with TaliPCApp (version 1.0). The percentage of apoptotic cells were exhibited as the annexin V-positive cells compared with the control group.

### 3.10. Statistical Analysis

All data measured from the three individual experiments were analyzed. The data were presented as mean ± S.E.M. Statistical significance was assessed using a Student’s T-test, with *p*-values less than 0.05 considered statistically significant.

## 4. Conclusions

The present study demonstrated that THC strongly prevents HT22 cell death induced by glutamate. Its protective mechanisms occur through the inhibition of the accumulation of intracellular ROS as an antioxidant property, blockage of MAPK activation, and prevention of apoptotic cell death of HT22 cells by glutamate. Therefore, THC may be useful as a potent neuroprotective metabolite of curcumin against glutamate-mediated neuronal death.
